# Trouble from the tiger: first detection of dengue virus in *Aedes albopictus* in Australia during a 2024 dengue outbreak on Masig Island

**DOI:** 10.1186/s13071-026-07553-4

**Published:** 2026-07-04

**Authors:** Mutizwa Odwell Muzari, Melissa C. Graham, Gerhard Ehlers, Rodney Bellwood, Joseph Davis, Dunstan Peniyamina, Gregor J. Devine, Brian J. Johnson

**Affiliations:** 1 Cairns Public Health Unit, Cairns and Hinterland Hospital and Health Services, Cairns, QLD Australia; 2 Mosquito Control Laboratory, QIMR Berghofer Medical Research Institute, Brisbane, QLD Australia; 3 Torres and Cape Public Health Unit, Torres and Cape Hospital and Health Services, Cairns, QLD Australia

**Keywords:** *Aedes albopictus*, Dengue virus, DENV3, Australia, *Aedes aegypti*

## Abstract

**Background:**

Dengue is not endemic in Australia and outbreaks occur occasionally after viraemic travellers visit parts of Queensland where dengue vectors are prevalent. Prior to 2016, *Aedes aegypti* was the primary vector of dengue in Australia. However, several dengue outbreaks have occurred more recently on islands in the Torres Strait region where *Ae. aegypti* has been displaced by *Aedes albopictus* following its first detection in the islands in 2005.

**Methods:**

In November 2024, adult female *Ae. albopictus* were collected during a dengue outbreak on Masig Island where *Ae. aegypti* is now absent after displacement by *Ae. albopictus.* Collected mosquitoes were pooled and screened for the presence of dengue virus by qRT-PCR and direct RNA sequencing.

**Results:**

Dengue virus RNA was detected by qRT-PCR in 9 of 12 mosquito pools. RNA sequencing of three positive pools confirmed the virus as belonging to dengue serotype 3 (DENV-3), consistent with the clinical serotype identified during the 2024 outbreak. Sequencing further detected four insect-specific viruses, including Bio Sioux River virus, reported in Australia for the first time.

**Conclusions:**

These results represent the first detection and whole genome sequencing of dengue virus in *Ae. albopictus* collected in Australia. The detection of DENV-3 RNA in *Ae. albopictus* during a concurrent outbreak of the same serotype in the absence of *Ae. aegypti* supports the ability of *Ae. albopictus* to drive independent dengue outbreaks. The findings confirm the risk of dengue outbreaks in mainland Australia if *Ae. albopictus* were to invade and expand beyond its current distribution in the Torres Strait.

**Graphical abstract:**

**Figure:**
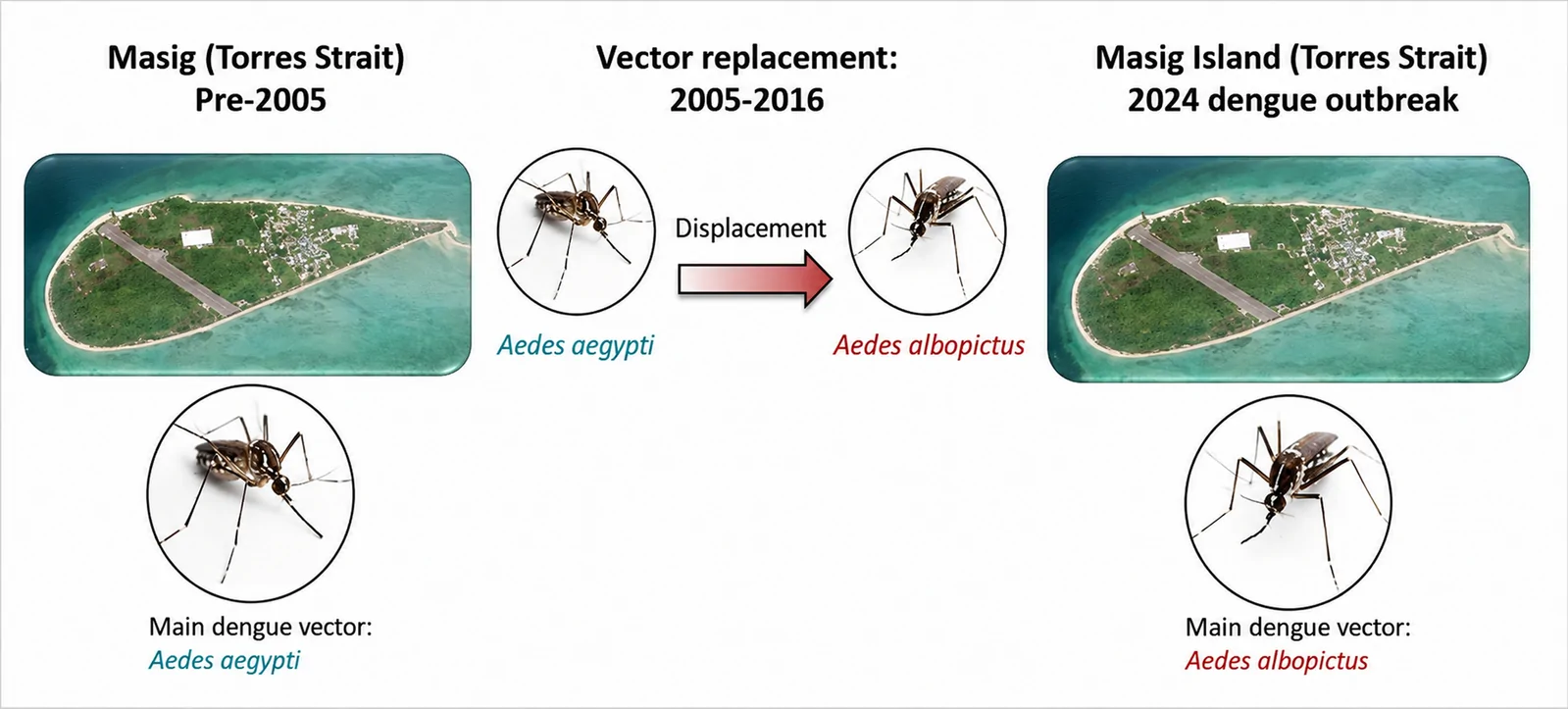

## Background

Dengue has emerged as the most important viral mosquito-borne disease globally with an estimated 390 million human infections per year worldwide. Dengue is caused by dengue virus (DENV), which has four different serotypes (DENV-1 to DENV-4), each of which are further subdivided into distinct genotypes [1, 2]. In Australia, dengue outbreaks have occurred frequently in parts of north Queensland, including Townsville, Cairns, and the Torres Strait islands, since the 1930s [3, 4] and occasionally in parts of central Queensland including Gladstone and Rockhampton [5]. These are areas where the vector, *Aedes aegypti,* has generally been abundant [3], however, Australia has no endemic transmission of dengue and outbreaks have normally been initiated by viraemic travellers returning from overseas [4–6]. Local transmission of dengue in parts of Northern Queensland has been ameliorated due to the successful *Ae. aegypti Wolbachia*-release strategy [7]. However, the arrival of *Aedes albopictus* on the mainland could reverse these gains and renew the threat of large dengue outbreaks and the possibility of dengue becoming endemic in Australia.

In 2005, established populations of *Ae. albopictus* (Asian tiger mosquito), a competent vector of dengue, chikungunya, and several other arboviral diseases [8], were detected for the first time on islands in the Torres Strait region of Australia [9]. An eradication campaign was rapidly established; although complete eradication was not achieved, the program successfully contained *Ae. albopictus* to the Torres Strait islands, preventing its establishment on the Australian mainland [10]. Since its detection, *Ae. albopictus* has rapidly spread to additional islands and has displaced *Ae. aegypti* on most islands in the Torres Strait [11].

**Fig. 1:**
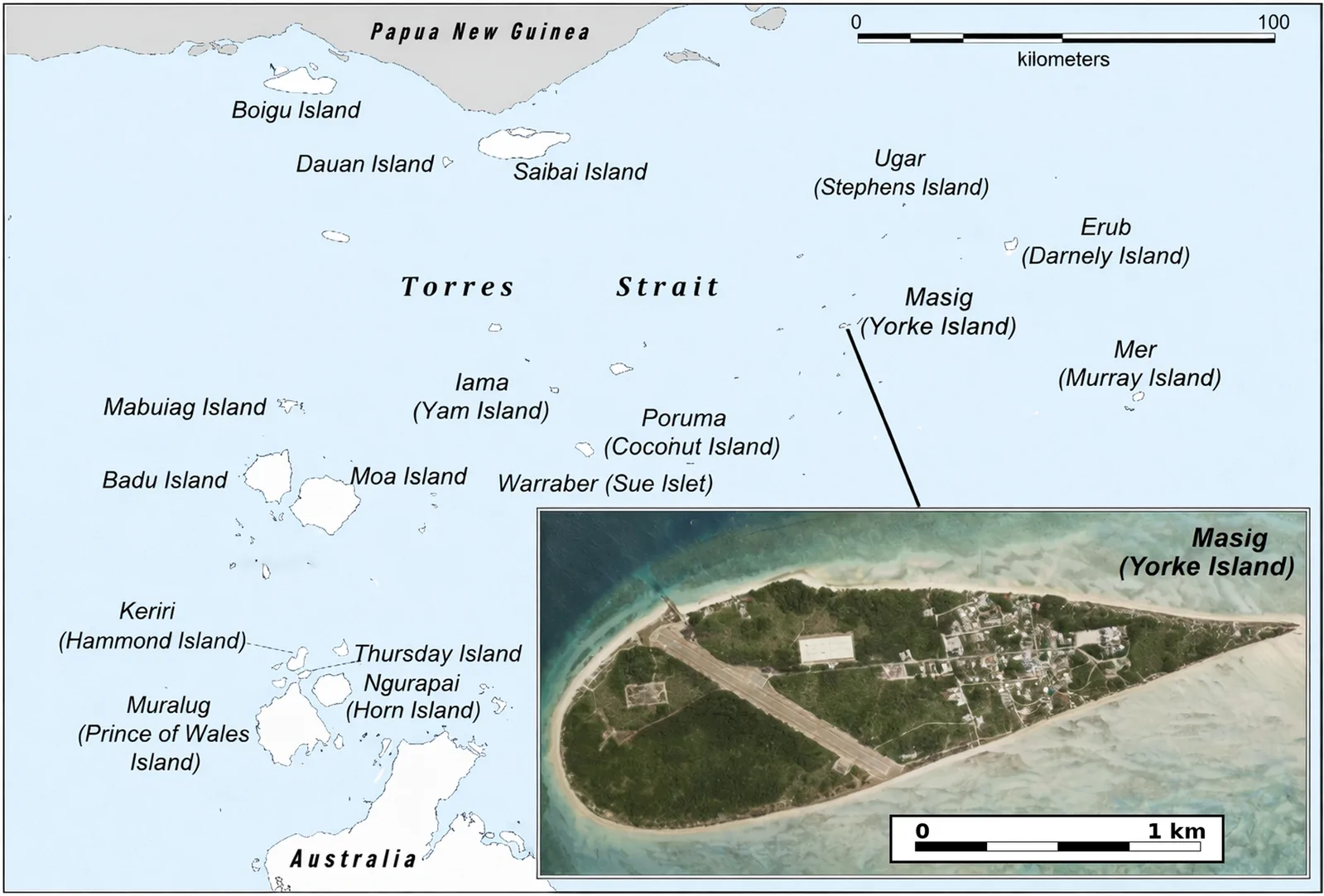
The Torres Strait region and aerial view of Masig Island (inset), showing the small community surrounded by forested areas suitable for high *Aedes albopictus* populations

From 2016, dengue outbreaks have occurred on some of the Torres Strait islands where *Ae. aegypti* has disappeared and *Ae. albopictus* has been the obvious suspect in local transmission [12, 13]. Despite extensive vector surveillance during these outbreaks, there had been no screening for presence of DENV in locally collected mosquitoes. This changed in November 2024, when field-collected *Ae. albopictus* females were screened and found to be positive for DENV-3 RNA during a DENV-3 outbreak on Masig Island (Fig. 1), where *Ae. aegypti* has been absent in surveys conducted in 2005, 2016, 2021 [9, 11, 14], and 2024 (Queensland Health, unpublished data).

Here, we describe the field and laboratory investigations that led to the first confirmed detection of DENV-3 RNA in Australian *Ae. albopictus*, undertaken as part of the coordinated public health response to the 2024 Masig Island outbreak, and discusses the implications of this finding for future dengue outbreak risks in the Torres Strait and mainland Australia.

## Methods

### Study site and mosquito collection

The Torres Strait region is the northernmost part of Queensland, Australia, stretching across the Arafura Sea up to the border with Papua New Guinea and consists of at least 100 islands of which 17 are inhabited [9]. Masig Island (also known as Yorke Island) is located among the islands in the central part of the Torres Strait (Fig. 1). It has ca. 270 residents in a community surrounded mostly by dense vegetation which harbours high populations of *Ae. albopictus* [14]. There have been no detections of *Ae. aegypti* on Masig Island in recent years [11, 14]. In November 2024, a dengue outbreak was declared on the island, eventually resulting in seven confirmed cases of DENV-3, and one probable case [13]. Local transmission ceased within 2 weeks due to intervention with harbourage spraying targeting *Ae. albopictus* habitat [15].

Prior to commencement of harbourage spraying, the state health vector control team collected adult mosquitoes using the sweep-net method in forested areas on the fringes of the community and adjacent to residential addresses of some of the positive cases. Mosquitoes were identified using morphological keys and preserved in ethanol for storage and transport.

### Dengue virus screening

Forty *Ae. albopictus* females were collected from Masig Island during surveillance efforts. An additional 32 male *Ae. albopictus* were also collected but these were not screened for the presence of virus. Collected female mosquitoes were divided into 12 pools of no more than six mosquitoes per pool. Pools were then homogenized (Beadbeater, Biospec Products Inc.) in 300 μl of DNA/RNA Shield (Zymo Research) in a 2 ml microcentrifuge tube. Total RNA was subsequently extracted from the homogenate using Quick-DNA/RNA Pathogen MagBead (Zymo Research), following the manufacturer’s protocol including Proteinase K and DNAase treatment and eluted in 50 μl. Pools were then screened for the presence of DENV by qRT-PCR using a pan-dengue assay targeting the conserved 3’ UTR region [16]. A positive control and 5-point standard curve generated from a DENV-2 isolate (New Guinea) was included in all tests. All tests were performed using the Luna Universal Probe One-Step qRT-PCR kit (New England Biolabs) and conducted in 25 μl reaction mixture containing 3 μl of total RNA, 1 × reaction mix, 600 nM of each primer, and 200 nM of each probe. The one-step qRT-PCR assay was performed twice in duplicate. The cycling conditions for all primer sets were 50 °C for 10 min, 95 °C for 2 min, followed by 40 cycles at 95 °C for 10 s, 52 °C for 10 s and 60 °C for 30 s. Negative template controls (NTC) consisted of water as a template. A sample was defined as positive if the average cycle threshold (C_t_) of the sample replicates was above 10 cycles and below the NTC if amplification of the NTC occurred prior to the assay cycling threshold (40 cycles). Amplicons were purified using the DNA Clean & Concentrator-5 kit (Zymo Research), according to the manufacturer’s instructions. The purified PCR products were Sanger sequenced with 3.4 pmol of DENV 3’ UTR reverse primer and 5 μl of purified amplicon in 10 μl. Cycle sequencing was performed using the BigDye Terminator v3.1 Cycle Sequencing Kit, purified using the BigDye XTerminator Purification Kit and loaded on a Bioanalyzer 2100 (Applied BioSystems).

### Total RNA sequencing

RNA extracts were quantified and RIN-scored with HS-RNA Tapestation on a Bioanalyzer 2100 (Agilent Biosystems). To increase RNA quantity, mosquito homogenates were re-extracted with the above protocol and pooled. Three samples that were positive for Sanger sequencing were run individually and the remaining nine samples were pooled in another two samples. Samples were run alongside other RNA for an unrelated project to increase RNA diversity in the final library. Library input was 10–100 ng of RNA using Illumina Stranded Total RNA Prep with Ribo-Zero Plus (Illumina, Inc.). A secondary depletion step, incorporating ribosomal RNA sequences from *Ae. aegypti* and *Ae. albopictus* (oPools, Integrated DNA Technologies) was spiked in at 2 μl of 50 pmol. Library was run on the NextSeq 2000 (Illumina, Inc.) using a P1 flow cell, 2 × 150 XLEAP-SBS chemistry. Fastq reads were quality trimmed and adaptors removed using BBDuk Trimmer (v38.84 by Brian Bushnell). Reads were merged and paired using BBMerge and Geneious v2025.2.1. A BLASTN (version 2.2.25+) search using command line (Mac OS) was performed against NCBI complete virus genome database: ref_viruses_rep_genomes (accessed 2024 and 2025). Reads were compiled and aligned against reference DENV3 genome (NC_001475) using Geneious. Fasta files were also BLASTX searched using DIAMOND; DAA files mapped using MEGAN (Daniel H. Huson, v6.25.10)[17]. For insect-specific viruses (ISVs), reads were compiled and run against reference genomes (Table 1).

**Table 1:** Insect specific viruses mapped from RNA sequencing data. Some of which had high genome coverage like AATV (85.5%). Most of these alignments had >99% sequence identity.

Virus Identity	NCBI	% Ref Seq Coverage	# of Reads	% Identity
Australian *Anopheles* totivirus	NC_035674	85.5	1097	92.3
Mac Peak Virus	NC_035187	16.6	281	100
Casuarina virus	NC_023986	32.4	511	99.5
Big Sioux River virus	NC_035184	23.4	434	98.8

## Results

### Dengue detection and total RNA sequencing

**Fig. 2:**
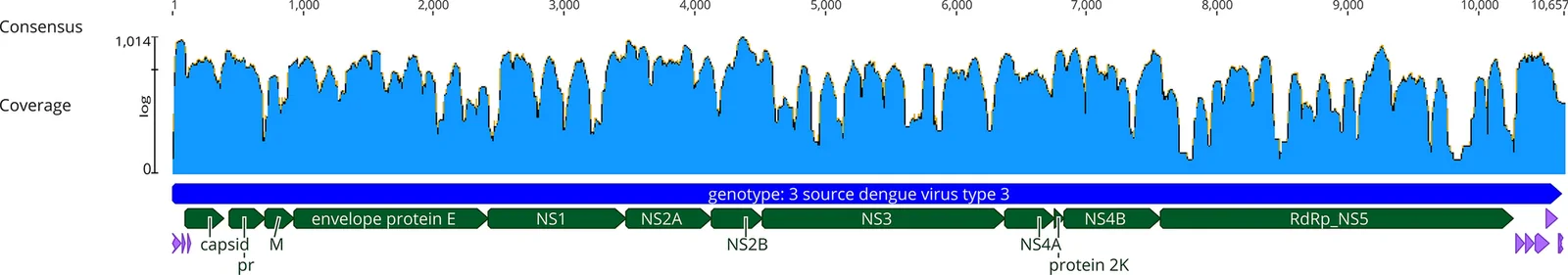
Alignment of blastn reads to dengue virus-3 complete genome NC_001475 using Geneious resulting in 99% of complete genome sequenced and 98.0% similarity to the reference genome

Nine out of 12 pools as well as the storage supernatant tested positive for DENV RNA by qRT-PCR. The C_t_ value of positive pools ranged from 23.8 to 32.3, with a mean C_t_ of 29.3 (95% CI 26.9–31.6). Amplicons produced from positive pools were sequenced and three samples returned good quality sequences, aligning to DENV-3 (NC_001475) (Fig. 2). Total RNA sequencing of these three pools resulted in 5.9 to 31.5 million reads per sample. Reads were mapped back to DENV-3 resulting in 99.0% of the whole genome sequenced (10,533 of 10,655 bp) and 98% sequence similarity to DENV-3 (NC_001475).

**Fig. 3:**
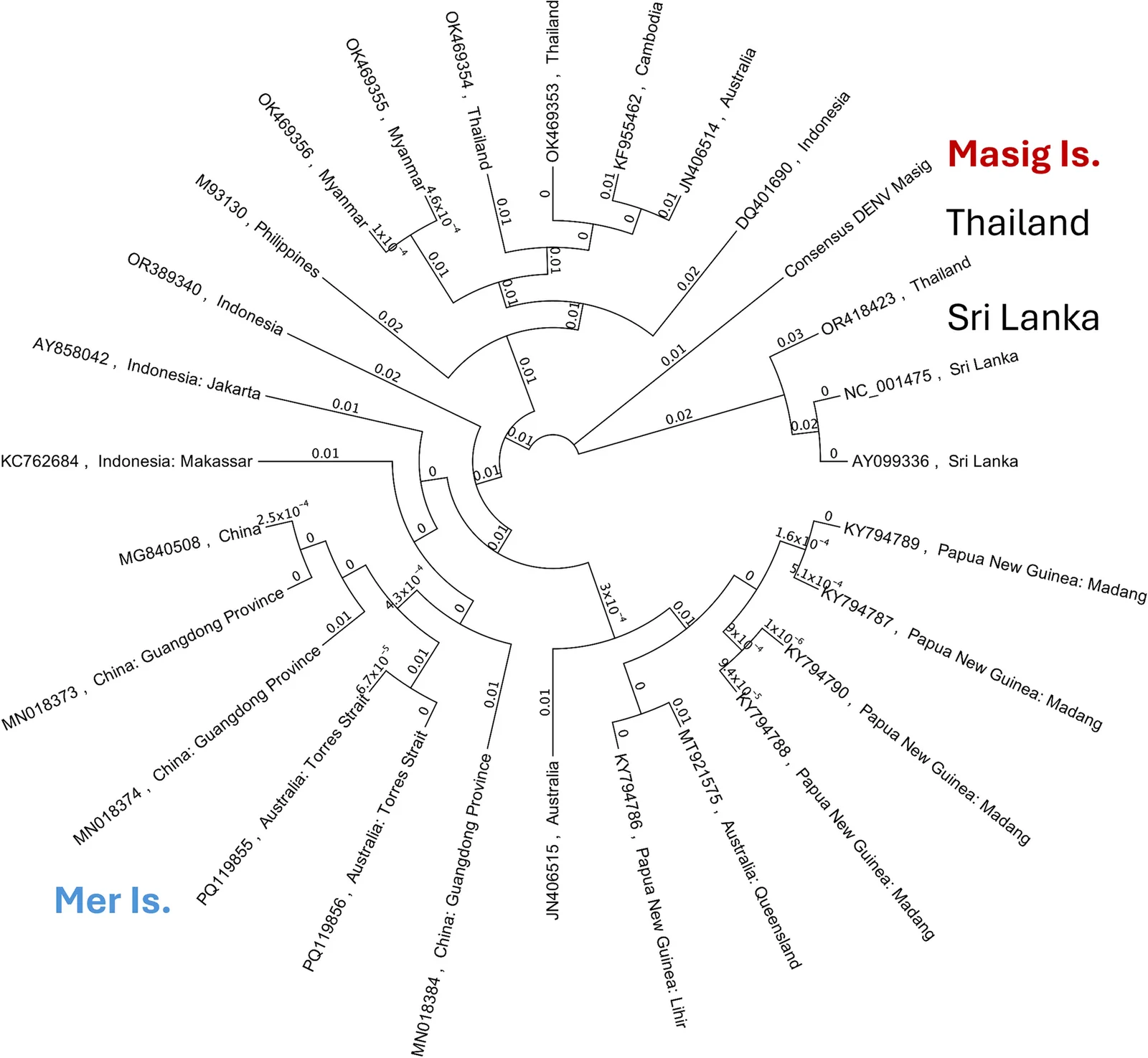
Phylogenetic tree for (27) DENV-3 strains: Australia, Papua New Guinea, Sri Lanka, China, Thailand, and Indonesia. Sequence obtained from *Ae. albopictus* mosquitoes collected on Masig Island (Consensus DENV-3 Masig) is most closely related to strains isolated from Sri Lanka (AY099336 and NC_001475) and Thailand (OR418423). The genome sequence obtained on Masig Island is not closely related to the two isolates obtained from human cases on neighbouring Mer Island (Torres Strait, Australia) during an earlier outbreak in 2024

A multiple sequence alignment was performed against 27 DENV-3 complete genomes isolated from human cases in Australia, Papua New Guinea, Sri Lanka, China, Thailand, and Indonesia. The DENV-3 sequence obtained from analysed *Ae. albopictus* pools (Consensus DENV-3 Masig) was most closely related to isolates from Sri Lanka (AY099336 and NC_001475) and Thailand (OR418423) than to the two isolates collected on neighbouring Mer Island in May 2024, suggesting the outbreaks were driven by separate viral incursions (Fig. 3). While there is no direct evidence of epidemiological links to these regions, frequent human movement within the Torres Strait and across the broader Indo-Pacific region provides a plausible mechanism for independent viral introductions, potentially via intermediate locations.

### Detection of other viruses

No RNA of other pathogenic viruses was detected from the screened pools, however, RNA sequencing produced sequences aligning to several ISVs. Four of these were mapped to their respective reference genomes with reads obtained ranging from just 281 to over 1000 (Table 1). This includes Australian *Anopheles* totivirus (NC_035674, 85.5%), a recently discovered orbivirus from Eastern Australia [18], for which nearly the whole genome was obtained. Casuarina virus (Mesoniviridae, discovered in 2014 in Australia) [19] and Mac Peak virus (Flaviviridae, discovered in 2017 in Australia) [20] (32.4% and 16.6% reference genome coverage, respectively) were also well characterised. Big Sioux River virus (NC_035184) was also detected.

## Discussion

The detection of DENV-3 RNA in *Ae. albopictus* collected during a concurrent outbreak of the same serotype on Masig Island corroborates the ability of *Ae. albopictus* to drive major dengue outbreaks under Australian conditions in the absence of *Ae. aegypti*. This result also incriminates *Ae. albopictus* as the key vector in other Torres Strait islands where dengue outbreaks occurred following the displacement of *Ae. aegypti.*

The role and impact of *Ae. albopictus* in dengue transmission tends to vary among geographical locations. For example, in parts of India and Taiwan, *Ae. albopictus* has been reported to be a secondary vector in outbreaks largely driven by *Ae. aegypti* [21, 22]. On the other hand, *Ae. albopictus* has been the primary vector of large dengue outbreaks in Macao [23], Guangzhou [24], and Tokyo [25]. It is reasonable to expect that *Ae. albopictus* could cause similar large outbreaks in Australia.

The relatively high proportion of DENV-positive mosquito pools observed in this study may, in part, reflect the unique ecological and epidemiological context of the Torres Strait islands. These settings are characterized by confined geography and reduced host and mosquito diversity [26, 27]. On Masig Island, the limited availability of alternative, non-human vertebrate hosts, together with the dominance of *Ae. albopictus* in the absence of *Ae. aegypti* [11], likely creates conditions conducive to intense focal transmission, thereby increasing the likelihood of detecting infected mosquitoes compared to more heterogeneous mainland environments. Furthermore, confinement of mosquito collections close to addresses of confirmed human cases during the outbreak may have contributed to the relatively high proportion of DENV-positive mosquitoes.

Although detection of viral RNA alone does not confirm the presence of infectious virus, our total RNA sequencing data provides additional support for biologically meaningful infection. Reconstruction of approximately 99% of the DENV genome from positive samples is unlikely to result solely from fragmented or degraded RNA and is more consistent with the presence of abundant viral RNA as expected during active replication within the mosquito host. Nonetheless, given that specimens were preserved in ethanol, precluding virus isolation, these findings should be interpreted with appropriate caution.

The outbreak on Masig Island was likely to increase beyond the initial eight cases, but dengue transmission ceased early [13] due to early detection, the small size of the island, and rapid inter-agency response incorporating an effective vector control strategy against *Ae. albopictus* [15]*.* In contrast, an outbreak of up to 74 cases on neighbouring Mer Island was detected relatively late, some 14 weeks from the first probable case, and continued until a week-long intervention was completed within 5 days of the first formally notified case [12].

The observations from Mer and Masig Islands demonstrate the enormous risk posed by *Ae. albopictus* if this species were to successfully expand onto mainland Australia, where several high population cities have been identified as having climatic conditions suitable for its establishment [28]. Control of dengue outbreaks caused by *Ae. albopictus* on the Australian mainland would not be as effective as have been recorded on the islands due to the vastness and complexity of the areas at risk and greater connectivity of major population centres, factors that have attributed to the failure to control or limit the spread of this species in other countries [29–31].

In recent years, local transmission of dengue on the Australian mainland has been greatly reduced due to the successful *Wolbachia*-release strategy against *Ae. aegypti* in parts of northern Queensland [7]. However, the arrival of *Ae. albopictus* on the mainland could reverse these gains and renew the threat of large dengue outbreaks and the possibility of dengue becoming endemic in Australia. The threat of *Ae. albopictus*-driven dengue transmission on the mainland underscores the importance of sustained political and financial support to maintain and strengthen the current strategies that minimise the risk of this species expanding beyond the Torres Strait [15]. Beyond dengue, of the four concurrently identified ISVs, Big Sioux River virus is reported here for the first time in Australia. Although ISVs are not associated with vertebrate disease^32^, ^33^, there is increasing evidence to suggest that ISVs can interact with pathogenic arboviruses, either through superinfection or competitive interference [34–36], thereby possibly modulating mosquito vector competence and shaping local transmission dynamics. The significance of this potential interaction requires further investigations.

## Conclusions

Our findings confirm that *Ae. albopictus* can drive dengue outbreaks under Australian conditions in the absence of *Ae. aegypti* and demonstrate the critical importance of rapid detection and targeted control responses. The contrasting outbreak trajectories on Masig and Mer Islands in 2024 highlight the consequences of delayed intervention, while climatic suitability across major mainland cities underscores the substantial risk should *Ae. albopictus* expand beyond the Torres Strait into mainland Australia. Such an incursion could erode gains achieved through *Wolbachia*-based replacement strategies targeting *Ae. aegypti* and increase the likelihood of large dengue outbreaks. These results reinforce the importance of maintaining strong surveillance and biosecurity measures to prevent *Ae. albopictus* establishment on the mainland.

## Data Availability

The consensus genomic DENV-3 sequence generated as part of this study is available at NCBI GenBank (accession: PZ516052).

## Abbreviations

qRT-PCR: Quantitative real-time polymerase chain reaction
DENV: Dengue virus
RNA: Ribonucleic acid
DNA: Deoxyribonucleic acid
ISV: Insect specific virus
RIN: RNA integrity number
HS: High sensitivity
